# Splenic Angiosarcoma with Bone Marrow Involvement Initially Diagnosed as Systemic Mastocytosis: A Case Report

**DOI:** 10.7759/cureus.5804

**Published:** 2019-09-30

**Authors:** Paul Plantinga, Sadaf Rahman, Kamilia Rizkalla, Jessica G Shepherd, Chai W. Phua

**Affiliations:** 1 Pathology and Laboratory Medicine, Schulich School of Medicine & Dentistry, Western University, London, CAN; 2 Medicine, Schulich School of Medicine & Dentistry, Western University, London, CAN; 3 Pathology and Laboratory Medicine, London Health Sciences Centre, London, CAN; 4 Pathology and Laboratory Medicine, London Health Science Centre, London, CAN; 5 Hematology, London Health Sciences Centre, London, CAN

**Keywords:** splenic angiosarcoma, bone marrow, autopsy

## Abstract

We describe the case of a 67-year-old female patient presenting with constitutional symptoms and rapid decline. Two bone marrow core biopsies were performed, with spindled cells identified and thought to represent marrow involvement by systemic mastocytosis on the first biopsy. A diagnosis of metastatic vascular malignancy with sarcomatoid features was favored on the second core biopsy. The patient rapidly deteriorated and passed away. The post-mortem examination revealed a splenic angiosarcoma with metastasis to the liver and bone marrow. Splenic angiosarcoma is a rare, aggressive entity, with bone marrow metastasis even more uncommon. This report perceives this as a diagnostic consideration on bone marrow biopsies with spindled cells and explores the diagnostic dilemma and overlapping features of systemic mastocytosis and angiosarcoma.

## Introduction

Primary splenic angiosarcoma is a rare, aggressive vascular malignancy with a dismal prognosis. It is composed of malignant endothelial cells with variable morphology, and can often be spindled [1-4]. The use of immunohistochemistry is required to make the diagnosis; however, unexpected immunohistochemical staining patterns can lead to diagnostic difficulty. We describe the case of metastatic primary splenic angiosarcoma masked by preliminary diagnoses of idiopathic myelofibrosis and systemic mastocytosis. This report explores the diagnostic dilemma and overlapping features of systemic mastocytosis and angiosarcoma.

## Case presentation

A previously healthy 67-year-old female presented to a community hospital with a four-month history of rapidly progressive decline with early satiety, weight loss, and night sweats. There was no significant family medical history. Physical examination revealed palpable hepatosplenomegaly, bilateral pitting edema of the lower limbs, scleral jaundice, and pallor. Additional workup was significant for hepatosplenomegaly (liver was 25 cm and spleen 18 cm on CT), and innumerable hypodense lesions of liver, spleen, and bones. Peripheral blood demonstrated leukoerythroblastic features and mild thrombocytopenia. She underwent an urgent bone marrow biopsy, which reported a preliminary diagnosis of idiopathic myelofibrosis. To confirm the diagnosis of myelofibrosis, the case was referred to a tertiary center for evaluation, including a review of the bone marrow aspirate and core biopsy, and testing for driver mutations associated with primary myelofibrosis. An urgent outpatient hematology-oncology referral was also made. 

Unfortunately, the patient deteriorated quickly with progressive liver dysfunction, anasarca, and transfusion-dependent bicytopenia. She was then transferred to a tertiary care center and admitted to the hematology-oncology ward for further management. Working diagnosis at that time included primary myelofibrosis with significant extramedullary hematopoiesis causing hepatosplenomegaly. Laboratory investigations at presentation in our center were pertinent for the following (normal reference ranges given in parentheses): total bilirubin: 72.7 μmol/L (3-22 μmol/L); direct: bilirubin 21.4 μmol/L (0-5 μmol/L); hemoglobin: 69 g/L (115-160 g/L); total leukocytes: 9 x 109/L (4-10 x 109/L); platelets: 21 x 109/L (150-400 x 109/L); nucleated red blood cells: 10-20/100 leukocytes (0/100 leukocytes); INR (international normalized ratio): 3.1 (0.9-1.1); fibrinogen: 0.33 g/L (1.7-5.9 g/L); ALT (alanine aminotransferase): 104 U/L (9-52 U/L); AST (aspartate aminotranferase): 241 U/L (14-26 U/L); ALP (alkaline phosphatase): 171 U/L (38-126 U/L); LDH (lactate dehydrogenase): 750 U/L (<215 U/L); JAK2 v617F mutation: negative; next-generation sequencing for myeloid disorders revealed no variants; hepatitis B immune from vaccination; and non-reactive for EBV (Epstein-Barr virus) IgM, CMV (cytomegalovirus) IgM, hepatitis C antibody, and HIV (human immunodeficiency virus) antibody.

**Figure 1:**
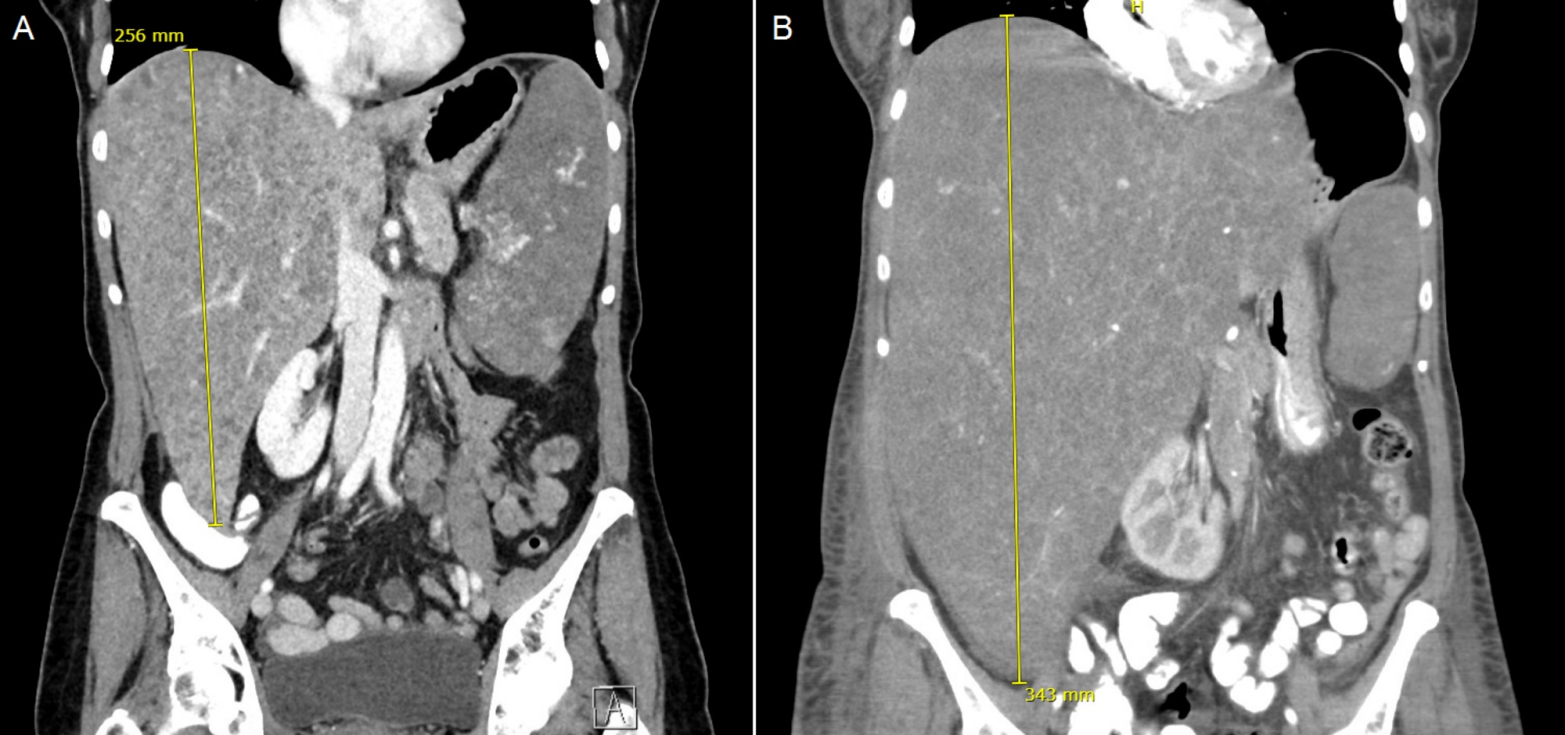
Coronal CT image of the abdomen with massive hepatomegaly. The liver measured 25.6 cm in craniocaudal length on initial imaging (A), and had enlarged to 34.3 cm on subsequent imaging five weeks later (B), displacing the right kidney and with mass effect

A repeat CT revealed further enlargement of the liver (34.3 cm) causing a significant mass effect with a displacement of the right kidney (Figure 1). There were also several lucent bone lesions concerning for lytic bone lesions. Clinically, the patient became more edematous and her liver enzymes rose briskly. As the rapidity of her deterioration was out of keeping with extramedullary hematopoiesis alone and the initial bone lesions were uncharacteristic for primary myelofibrosis, we pursued a transjugular liver biopsy, which was unsuccessful, and a repeat bone marrow biopsy.

The patient clinically deteriorated further and, on the sixth day of admission, was transferred to the intensive care unit for hypotension requiring vasopressors and multi-organ failure. Unfortunately, she passed away within 24 hours of her transfer. Pathologic results from her subsequent bone marrow biopsy and autopsy are reviewed below.

**Figure 2:**
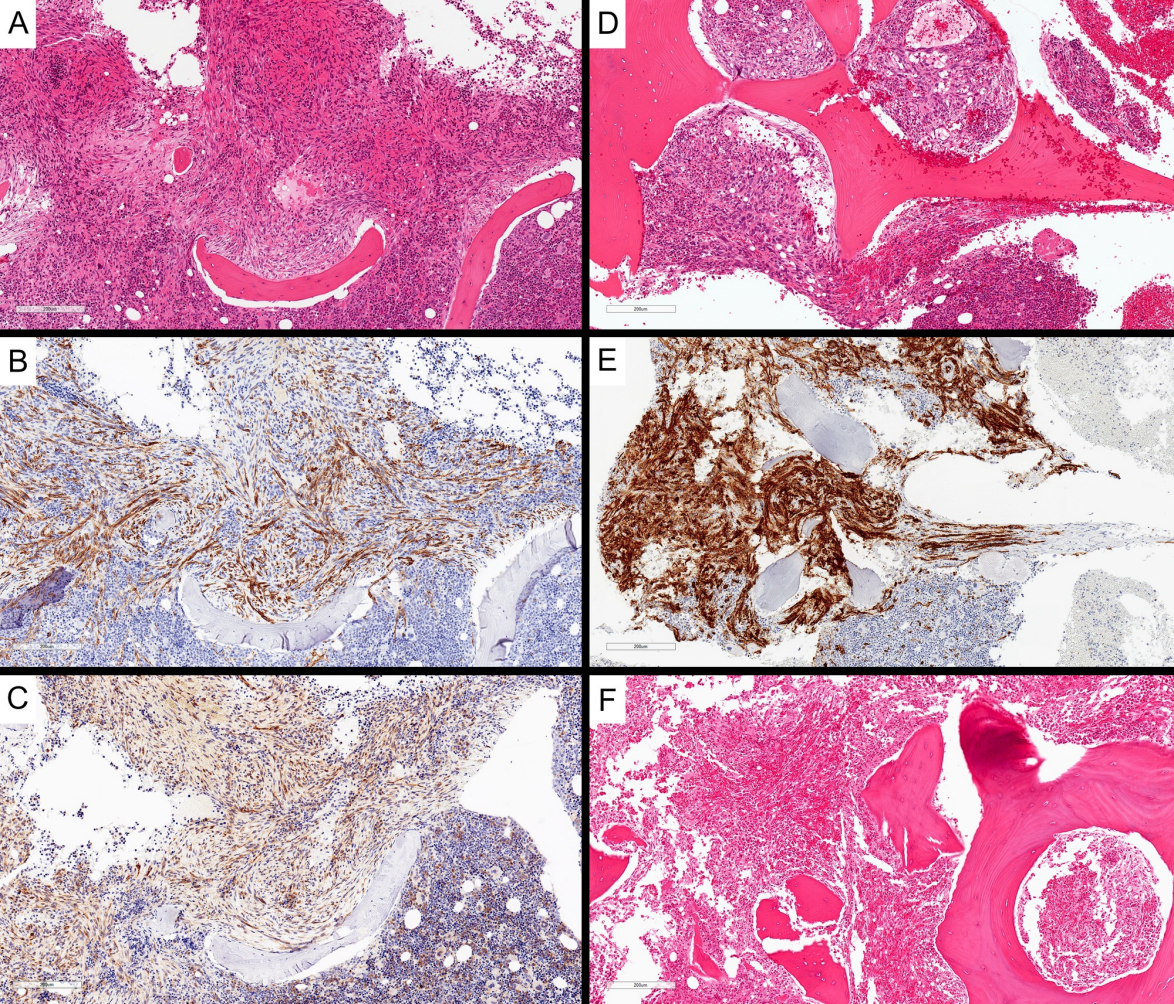
Bone marrow involvement by angiosarcoma. Initial bone marrow core biopsy showed spindled cells within the marrow, interpreted as atypical spindled mast cells (A, H&E, 100x magnification), with patchy CD34 staining (B, 100x magnification) and expressing weak CD117 (C, 100x magnification). Subsequent bone marrow core biopsy again showed atypical spindled cells (D, H&E, 100x magnification) with stronger CD34 expression (E, 100x magnification). Vertebral bone marrow from post-mortem examination demonstrated involvement by angiosarcoma (F, H&E, 100x magnification) H&E: Hematoxylin and eosin

Intriguingly, the secondary review of her initial bone marrow biopsy revealed a hypercellular marrow together with infiltration of spindled cells (Figure 2A) that were uniform and bland, admixed with relatively normal bone marrow elements. Immunohistochemistry stains showed positivity for CD34 and CD117 (c-KIT) (Figure 2B-C). Staining for CD2 and tryptase was not available through our lab, and CD68R and FLI-1 staining was not completed. The cells were negative for cytokeratin CK AE1/AE3. These spindled cells were interpreted as spindled mast cells due to morphologic features and CD117 positivity. Given the findings of multifocal dense infiltrates of presumed mast cells, of which greater than 25% were spindled, the diagnosis of systemic mastocytosis by WHO 2016 criteria was suggested. The subsequent bone marrow core biopsy (Figure 2D-E) was performed shortly before the patient's death and was reported after her death. On this biopsy, there were occasional foci of infiltrative vascular neoplasm with sarcomatoid features. There were hyperchromasia and pleomorphism in the spindled cells, and vascular spaces were identified. The neoplastic cells were positive for vascular markers CD34 and CD31, along with vimentin. Interestingly, in this biopsy, CD117 appeared negative. Reticulin fibers were increased within the infiltrative metastatic foci.

**Figure 3:**
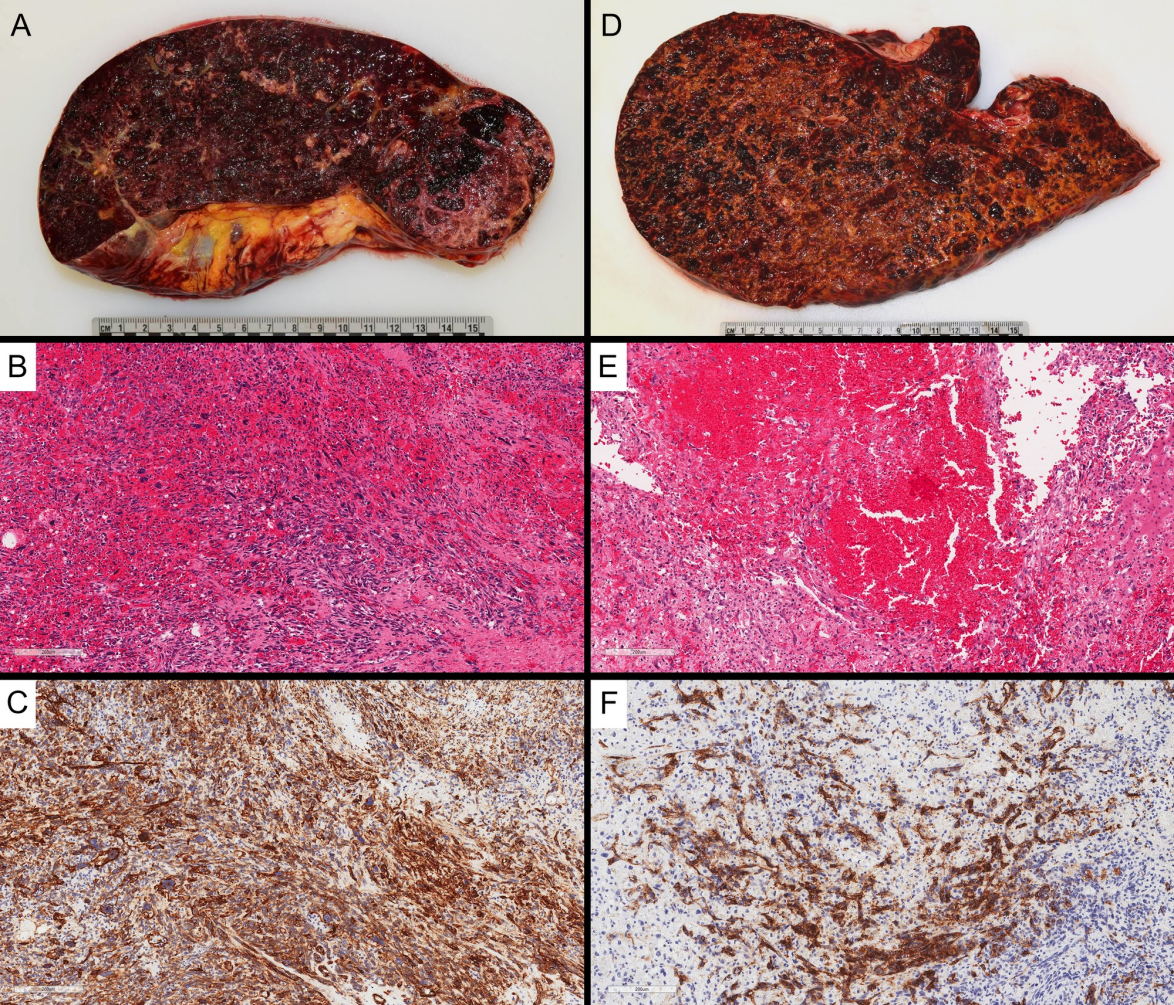
Post-mortem examination of the spleen (A) and liver (D) showed diffuse involvement by angiosarcoma, with minimal residual normal parenchyma, and diffuse enlargement of both organs. The diffuse involvement by malignant spindled cells is demonstrated histologically in the spleen (B) and liver (E) (H&E, 100x magnification), with diffuse positivity for CD34 by immunohistochemistry [C (spleen) and F (liver), 100x magnification] H&E: Hematoxylin and eosin

Macroscopic autopsy findings included massive hepatosplenomegaly (liver: 4,674 g, spleen: 1,364 g), with the parenchyma of both liver and spleen almost entirely replaced by diffuse, infiltrative, hemorrhagic neoplasm (Figure 3A, D). No definite discrete mass was identified. There was 650 mL of sanguineous fluid in the peritoneal cavity and diffuse hemorrhage in the retroperitoneum. There were no significant abnormalities in the remaining abdominal and pelvic organs. Microscopy revealed a poorly differentiated angiosarcoma. This was diffusely involving the spleen (Figure 3B-C) and liver (Figure 3E-F), with patchy involvement of vertebral bone marrow sections (Figure 2F). The lesion was composed of atypical, mitotically active, spindled cells, forming vascular channels with abundant background hemorrhage. The lesional cells were positive for vascular immunohistochemical markers CD34 and CD31 and negative for cytokeratin CK AE1/AE3. The eventual diagnosis was favored to represent primary splenic angiosarcoma with metastasis to the liver and bone marrow.

## Discussion

Primary splenic angiosarcoma is a rare, highly aggressive vascular malignancy comprising about 2.6% of angiosarcomas, with an incidence of approximately 0.14-0.25 cases per million individuals [1,5]. It is often metastatic to the liver, lungs, lymph nodes, and bone and has a poor prognosis [1-4]. Bone marrow metastasis from primary splenic angiosarcoma is very rare, with only a few reports in the literature [5]. The lesion is composed of abnormal, malignant endothelial cells, with variable histology between cases. The malignant endothelial cells can be round, polygonal, spindled, or epithelioid, forming vascular channels in well-differentiated areas, which are less defined in more aggressive diseases. Papillary projections into the vascular lumen and multilayering of the malignant cells can occur. In more poorly differentiated areas, there may be sheets of malignant cells, with hemorrhage and necrosis [1].

Immunohistochemistry is vital in making the diagnosis of angiosarcoma, typically with endothelial marker expression, including CD34, CD31, factor VIII related antigen, vascular endothelial growth factor (VEGF), and FLI-1 [6]. Occasionally, angiosarcomas can express cytokeratins, leading to difficulty in distinguishing from a poorly differentiated carcinoma [1]. The expression of CD117 has also been demonstrated in angiosarcoma [7,8]. It is unclear as to why CD117 expression differed between the two bone-marrow core biopsy samples. It could have been due to a sampling issue, localized expression in parts of the tumour, or related to the staining procedure.

Angiosarcoma involving the bone marrow can be mistakenly interpreted as systemic mastocytosis with bone marrow involvement. Mastocytosis is characterized by the accumulation and aggregation of neoplastic mast cells within one or more organ systems. The bone marrow is involved in nearly all cases [9]. Atypical morphology of the mast cells, particularly spindling, is included in the criteria for systemic mastocytosis. Neoplastic mast cells typically are immunoreactive for CD117, with an activating point mutation of KIT [9,10].

Systemic mastocytosis typically involves the bone marrow, whereas angiosarcoma involving the bone marrow is rare. Though these diseases are not classically within the same differential diagnosis, both can show spindled morphology, and both can express CD117 by immunohistochemistry. However, CD117 positivity is more typically seen in systemic mastocytosis and is less frequent in angiosarcoma. In addition, immunohistochemical staining patterns for both CD34 and CD117 can be challenging to interpret in the context of bone marrow biopsies, as blasts and immature precursors can show positivity for these markers. CD34 expression is typically absent in neoplastic mast cells but can be displayed on their precursor neoplastic stem cells [9]. Notably, CD31, while typically positive in angiosarcoma, has been found to be negative in systemic mastocytosis [10]. A summary of pathological findings of systemic mastocytosis compared with splenic angiosarcoma is given below (Table 1).

**Table 1:** Summary and comparison of pathological findings in systemic mastocytosis and splenic angiosarcoma

	Systemic mastocytosis	Splenic angiosarcoma
Sites involved	Bone marrow, spleen, skin, liver, gastrointestinal tract	Spleen, with metastasis to liver, lungs, lymph nodes, and bone, rarely to bone marrow
Morphology	Clusters of mast cells, which may show cytologic atypia, including spindling and poly-lobed nuclei; may show associated eosinophilia	Variable pleomorphic, malignant nuclei: may be rounded, polygonal, fusiform, or epithelioid; vascular spaces in well-differentiated cases
Immunohistochemistry	Positive for CD117, CD2, CD25, tryptase; CD31 and CD34 usually negative	Positive for vascular markers CD31, CD34, FLI-1, VEGF; rarely CD117 positive; epithelioid variants may be cytokeratin positive

## Conclusions

This case underscores the diagnostic dilemma that can arise in differentiating angiosarcoma from systemic mastocytosis, both uncommon diseases, based on initial bone marrow biopsy. Spindled lesions involving the bone marrow, with an unusual morphology and immunohistochemical staining patterns, can present significant diagnostic challenges, and rare entities should be considered and thoroughly investigated.
